# COVID-19: Yin and Yang and Herd Immunity

**DOI:** 10.1017/dmp.2020.229

**Published:** 2020-12

**Authors:** James J. James

Yin and yang is a Chinese concept of dualism, describing how seemingly opposite or contrary forces may actually be complementary, interconnected, and interdependent. Application of this concept to the coronavirus disease (COVID-19) response may better enable us to simultaneously optimize reducing the negative medical and socioeconomic impacts of the pandemic.

To date (June 29, 2020), there have been over 10 000 000 “cases” of COVID-19 reported with 500 000 deaths in some 213 countries across the globe.^1^ In response to this pandemic, a myriad of “experts” have published over 25 000 COVID-19-related papers^2^ on every conceivable aspect of the disease based mostly on anecdote, opinion, and informed guesstimates. This has predictably resulted in often conflicting conclusions with the result that any policy put forth to control the pandemic can find support in the scientific literature. Given this backdrop, it is easy to understand the current divisiveness of opinions rampant across the United States: continued lockdowns to save lives versus the opening of the country now to prevent economic collapse; masks versus no masks; new cases are due to increased exposure versus increased testing; a second wave versus a prolonged first wave; achieving herd immunity needs a vaccine versus it can be effective in itself; and so forth. Before offering some suggestions on going forward, a brief review of a recent historical event might well serve as an instructive analogy to help move past our seeming paralysis of purpose.

The Great Chinese Famine that took place from 1959 to 1962 stands as one of the greatest man-made disasters in history with an estimated death toll due to famine numbering in the tens of millions. As with all disasters, this was a complex event with many factors contributing to the eventual outcomes. Unlike COVID-19, however, the root cause did not stem from nature but was a direct result of official policies introduced in the Great Leap Forward. In misguided and uninformed efforts to increase the yield of grain, farms were collectivized and the over-planting and deep plowing of shallow fields was officially promoted, resulting in a significant drop in crop yield. The counterproductive interventions were coupled with multiple conspiracy theories, accusations, and the widespread abuse of power at the regional and local levels by officials who themselves had ample supplies of food and drink and were more interested in currying political favor than the public good. False claims and exaggerations to give the illusion of having met quotas amplified the political inertia at the national level. Officials without any understanding of ecological principles launched a campaign to rid the country of sparrows that were blamed for the famine as they would feed on newly scattered seeds. This campaign was successful in decimating the sparrow population only to usher in an explosion of locusts that went on to totally devastate the grain crop. Finally, in 1961, the famine was officially recognized and a government field evaluation determined that the disaster was 70% due to human error. This led to the ending of the cultural revolution and the institution of sound corrective policies to include the importation of 250 000 sparrows from the Soviet Union.^3^

The response to COVID-19 in the United States to date has likewise been largely ineffective due to false claims, misinformation, uninformed decision-making, and, most of all, political posturing at the expense of public health. Most unfortunately, there are yet no sparrows in the form of an effective vaccine or pharmaceutical cure available to end our collective pain. Further, there are no obvious best answers on moving forward simply because we do not know and cannot predict the future. However, we can offer some considerations that should facilitate our decision-making regardless of the ultimate course of the pandemic and allow us to optimize our medical response and protect our socioeconomic infrastructure if we recognize these goals as complementary and not in conflict. This is not about lives versus dollars; it is about optimizing both the individual medical outcomes and overall population health. Factors to consider include:

1. *The virus is in charge*. We cannot contain it, and we currently cannot prevent nor adequately treat the disease it produces. We need to learn to live with COVID-19 as we have with a myriad of other infectious maladies.
2. *Cases*. The medical definition of a case is “an instance of disease or injury.” For COVID-19, we define a case by a lab test that shows the presence of a virus not the presence of an infection. We are home to an estimated 30 trillion plus microorganisms, many of which can be pathogenic, but we do not use them to define a disease. Further, significant differences in tests, testing protocols, numbers, and reporting make comparisons over time and across states practically useless. To better track the progress of COVID-19, we need to report hospitalizations, which would give us a more valid and standardized case definition that can provide the demographics needed to help drive targeted control measures in a meaningful manner.^4^ This simple measure would also greatly diminish the absurdist comparisons and exaggerated media claims that we are constantly exposed to, based on the overall number of test positives without regard to demographic or clinical characteristics.
3. *Risk and fear*. The extent of public fear around COVID-19 is real, palpable, and widespread. The question is whether the level of fear is warranted by the level of risk involved. This requires assessing an individual’s risk of having a terminal outcome. Given the multitude of variables that have to be considered, this can only be approximated at best. Based on real world data, a back of the envelope calculation gives an overall population risk of a fatal outcome of 0.04% with that risk significantly decreasing with younger age and markedly increasing for the older and those with comorbidities. Another concern that influences this equation is the fear of transmitting the virus to others, especially to elders, and that is very real; however, even in that age group, over 85% survive of those infected.^1^ Given these numbers, the fear level may not be warranted, but, once fear is instilled, it is difficult to dilute. Sadly, the only weapon at our disposal to combat fear is consistent health communication, and the information from various government levels is conflicting at best and anything but reassuring.
4. *Vaccines*. Hopes for the rapid development, production, and deployment of a safe and effective vaccine are high. They were just as high 40 years ago for an AIDS vaccine and over the past decade for a universal flu vaccine. However, given the nature and relative stability of the COVID-19 virus, there is a reasonable expectation for an effective vaccine, but this is not assured. Even with the resources provided under the Warp Speed initiative, it is highly doubtful that a safe and effective vaccine, in sufficient quantity, ready for injection, would be available within the next 12 months. Even if these obstacles could be overcome, recent surveys indicate that up to 70% of the population would be reluctant to accept the vaccine upon release.^5,6^ Compounding the overall conundrum is the relative non-effectiveness of vaccines in the elderly, the very group at the highest risk for COVID-19.
5. *Herd immunity*. Herd immunity is a basic concept of epidemiology and simply means you must have a certain number of susceptible individuals left in a population to continue the chain of transmission. Unfortunately, it has become quite controversial in relation to COVID-19. The reasons for this are (1) the term conjures up images of chicken pox parties with people purposely exposed to a potentially lethal disease, and (2) herd immunity only has application in conjunction with an available vaccine based on the erroneous idea that it is an all-or-none construct, as opposed to a continuous variable directly related to the number of non-susceptible individuals in the population. This simply means that, as the non-susceptible individuals increase, the transmission rate decreases (fewer cases) and we effectively flatten the curve. To determine the optimal level to interrupt transmission, we need to estimate the rate of transmission. Using traditional models and COVID-19 data, herd immunity levels are estimated to be between 60 and 80%. However, these models assume homogeneity of risk and exposure within the population and do not adjust for significant differences in risk by age and other variables that are prominent with COVID-19. A recently published paper adjusting for these characteristics shows the effective immunity level to be 43%, which is significantly below that previously assumed.^7^ Finally, herd immunity is not a strategy. It is a natural phenomenon that is the default mechanism for achieving interruption of viral transmission.
6. *Susceptibility*. Lockdowns are advocated most strongly by those who feel that almost 300 000 000 Americans remain fully susceptible to COVID-19. This estimate is highly suspect as accumulating data demonstrate a higher than expected prevalence of previously infected as well as a substantive number of relatively resistant individuals by age and/or genetic factors.^8^ Further, given the apparent ubiquity of the virus, it is hard to imagine that a large proportion of the population has not already been exposed without demonstrating any ill effects. However, supposition aside, what we do know is that there are very well-defined, high-risk groups by age, comorbidity, and institutionalization, all of which are particularly vulnerable to continued exposure to COVID-19. Rather than socioeconomically destructive universal lockdowns, it would seem most prudent to target interventions to those groups, as appropriate, while we await the arrival of the sparrows and gradually approach population herd immunity.

In the week that it has taken to prepare this editorial, the number of reported COVID-19 “cases” has risen dramatically in many states and for the country as a whole while at the same time the number of polymerase chain reaction (PCR) tests has had a similar rise. To put the numbers in perspective, there has been a 60% increase in test positives^1^ versus a 45% increase of tests performed.^9^ Given the continued use of a deficient case definition and incomplete hospitalization and demographic data, it is difficult to truly assess the continuing impacts of the pandemic when this information is critically needed to inform continued decision-making on reopening the economy and restarting the educational system. As a result, the friction between the yins and yangs continues and the public loses. If we expect the public to be “all in this together,” we can expect less from ourselves. It is time to realize that our goals are complementary, not oppositional, and to be guided by an observation made by President Truman, “It is amazing what you can accomplish if you do not care who gets the credit.”
